# Adolescents’ Views on Seeking Help for Emotional and Behavioral Problems: A Focus Group Study

**DOI:** 10.3390/ijerph17010191

**Published:** 2019-12-27

**Authors:** Suzanne J. van den Toren, Amy van Grieken, Marjolein Lugtenberg, Mirte Boelens, Hein Raat

**Affiliations:** Department of Public Health, Erasmus University Medical Center, 3000 CA Rotterdam, The Netherlands; s.vandentoren@erasmusmc.nl (S.J.v.d.T.); a.vangrieken@erasmusmc.nl (A.v.G.); m.lugtenberg@erasmusmc.nl (M.L.); m.boelens.1@erasmusmc.nl (M.B.)

**Keywords:** emotional and behavioral help-seeking behavior, adolescence, barriers and facilitators to help-seeking, help sources, internet help-seeking

## Abstract

This study aimed to get insight into adolescents’ views on help-seeking for emotional and behavioral problems. Fourteen focus groups were conducted. Two vignettes, depicting one healthy adolescent with few issues and one adolescent with severe psychosocial problems, were used to structure the focus groups. The focus groups were framed within a youth help-seeking model. Adolescents (mean age of 15.0 years) generally reported seeking help from friends or the internet for mild issues and from a person they trust like a parent or school mentor, for more severe problems. Adolescents correctly recognized the issues in vignette one as surmountable and the problems in vignette two as severe. A bond of trust with a help source was regarded as the main facilitator for the decision to seek help. Adolescents reported a preference for help sources who clearly displayed their expertise for the issue at hand and for informal help-sources, particularly friends.

## 1. Introduction

Adolescents aged 13–18 years demonstrate an increase in health risk behaviors such as drug use and unsafe sex, which may persist into adulthood [1,2,3]. Furthermore, adolescence is characterized worldwide by a decrease in psychosocial well-being, for example, a rise in mood disorders [4,5]. At the same time, adolescents are less likely to seek help for their concerns compared to adults [6] and compared to adolescents who do not experience these concerns [7]. Help-seeking behavior is considered important as it may reduce the persistence and severity of the issues for which help is sought [8,9,10,11,12]. To illustrate, a significantly lower prevalence of behavioral disorders was found for children and adolescents who consulted with their mother or friends about their issues [8]. In a study on the perceived benefits of seeking help for mental health problems among university students, the top three perceived benefits as reported by students were reduced feelings of stress, improved mental functioning, and the resolution of one’s problem [9]. 

Help-seeking is defined as the behavior of actively seeking help by communicating with informal (e.g., family and friends) and formal (e.g., professionals with a recognized role and appropriate training in providing help and advice) sources, to obtain help in terms of understanding, advice, information, treatment, and general support in response to problems or distressing experience [13]. Srebnik et al. developed a model for youth help-seeking and service utilization [14]. According to this model, there are three stages in the help-seeking pathway. The first stage is problem recognition. The second stage is the decision to seek help. In the third stage, the help source that youth turn to for help is considered. 

When looking at the stage of problem recognition, the first stage of the model by Srebnik et al., thus far, has found contrasting results. For example, in a study on mental health literacy among 1678 adolescents, less than 25% correctly recognized the problem in the displayed vignette as depression [15]. However, another study among 1002 adolescents found a correct appraisal of a clinical vignette depicting depression of more than 80% [16]. Regarding Stage 2, the decision to seek help, several studies demonstrated low intentions to seek (professional) help in adolescence [6,7,17]. Two-thirds of the adolescents did not seek professional help for their perceived difficulties with emotional and behavioral issues that were classified as abnormal [17]. Furthermore, in a survey study using clinical vignettes (i.e., a peer with a clinical disorder was portrayed) among more than 1000 high school students, one-third of adolescents failed to recommend this fictional peer to seek help for his/her depression or social anxiety [18]. Therefore, more insight into adolescents’ help-seeking for emotional and behavioral issues is of great importance to public health services. 

In relation to the third stage, considering the sources that youth turn to for help, previous research identified several informal (e.g., family and friends) and formal (health professional and teachers) sources of help [19]. In addition, the internet is increasingly used as a source of help, however, little is known about the online help-seeking behavior of adolescents [20]. Best et al. [21] found a difference between informal and formal online help-seeking pathways. Informal online help-seeking pathways in adolescent males reduced stigma and increased the opportunity for social support, but simultaneously reduced anonymity and control. In contrast, formal online help-seeking pathways increased anonymity, but concurrently, they also raised concerns on the abilities of adolescents to locate and assess the quality of the information correctly [21].

Previous studies provided a breadth of knowledge on help-seeking for mental health issues or other specific issues. Furthermore, most of these studies focused on a specific help source used a quantitative approach [6,7,8,10,22]. This qualitative study aims to add to previous studies by exploring in-depth interpretations into adolescents’ views on help-seeking for emotional and behavioral issues This study considered help sources and issues for help-seeking bottom-up with a broad focus on potentially all types of help sources and problems, including the internet as a relatively new and less well-studied source of help.

## 2. Materials and Methods 

### 2.1. Research Design

The present qualitative study used focus groups to explore adolescents’ views on seeking help for emotional and behavioral problems. Focus groups provided the opportunity to obtain detailed information through planned discussions; the researcher provided the focus and the data came from the group interaction. This was all performed in a non-threatening environment that facilitated the sharing of perceptions by participants [23,24].

This study was embedded within a larger study on the evaluation of the extension of preventative youth health care for adolescents. The Dutch youth health care system offers nationwide preventive health care through anticipatory guidance for children and youth to promote growth, development, and health. All children and adolescents were invited by the youth health care organization in their region to attend ‘preventive periodic health consultations’, which mostly took place at school with a physician or school nurse [25]. Previously, these consultations ended at the age of 13. Since 2013, an additional health consultation was offered to adolescents above the age of 13 years. With implementing this additional consultation, the need arose for evaluation. 

The Medical Ethics Committee of the Erasmus University Medical Centre Rotterdam declared that the Dutch Medical Research Involving Human Subjects Act (in Dutch: Wet medisch-wetenschappelijk onderzoek met mensen) did not apply to this research protocol and issued a declaration of no objection for this study. They gave permission to conduct this study and to submit the results for publication in a scientific journal in the future (MEC-2016-297).

### 2.2. Setting and Participants

The focus groups took place between March 2017 and February 2018. To select participants, 11 of the 25 Dutch youth health care regions represented by the National Office of Public Health and Safety (GGD GHOR the Netherlands) were asked to participate in the study. Six organizations that embodied a variety of regions agreed to participate in the qualitative part of the study. Each of these 6 organizations was asked to provide a contact person at 1 or 2 secondary schools in their region, depending on the variety of school levels that were offered. Six high schools agreed to participate in the focus groups. Other schools could not fit the participation in their schedule. The contact person at each secondary school then selected 1 or 2 classes and informed all students in these classes about the study either face-to-face or via e-mail. Adolescents could apply face-to-face or by sending an email to the contact person. An information letter about the study was then sent to interested adolescents and their parents, explaining to parents how they could object to the participation of their child. None of the parents objected to the participation of their child. We performed focus groups separately for (1) boys/girls and (2) pre-vocational education/senior secondary education. These education levels were both secondary education types. Pre-vocational education takes 4 years and prepares students for vocational education; senior secondary education takes 5 years and prepares children for higher professional education [26]. The focus groups were held at the location of the participating school during teaching hours in a private room.

### 2.3. Topic Guide

The topic guide included 2 vignettes. The 1st vignette depicted 1 adolescent with a few issues, who was insecure about intimacy-related issues with their partner. The 2nd vignette depicted an adolescent who had many issues, at home and in school, with high levels of school absenteeism. After one vignette was read, a discussion was started using pre-defined questions on the help-seeking behavior of the participants if they were to be in a similar situation, such as facilitating factors or barriers to seek help and the chosen help source (see Appendix A and Appendix B) [27,28]. Vignettes provided practical scenarios in an accessible form to the research population [27]. The method of using vignettes enabled the exploration of participants’ subjective belief systems: “Participants are typically asked to respond to these stories with what they would do in a particular situation or how they think a third person would respond” [28]. A vignette gave the opportunity to comment on other people’s situations in a less threatening manner compared to commenting on one’s own situation. In addition, it encourages participation and allows for exploration of actions in context [29].

Before the start of the focus groups, the topic guide was reviewed by 2 experienced health care professionals to see if the vignettes were a realistic rendition of adolescents and if the probes suited the purpose of this study. Adjustments were made accordingly. A trial focus group was held with 5 youth health researchers. The topic guide was adjusted following the recommendations from this trial focus group, for example, by providing more opportunities to ask questions and by including a broader explanation of the study. 

### 2.4. Focus Groups

The first author (ST) moderated all focus groups, and on 4 occasions, a research assistant was present. The moderator introduced the study and the purpose of the focus groups and informed the participants that the focus group would be recorded, that their names would remain confidential, and that they could stop their participation at any time. Adolescents provided written, informed consent for participation in the study. At the end of the focus groups, participants were asked to fill out a short questionnaire to acquire data on their age, sex, and education level, for which they also provided their written consent. If desired, participants received a coupon of 7.50 euros for their participation. The focus groups lasted between 20 and 49 min (35 min on average).

### 2.5. Data Analyses

**Figure 1:**
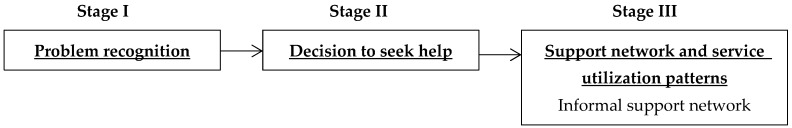
The three Stages of the help-seeking pathway from the model for youth help-seeking and service utilization adapted from Srebnik et al. [14].

Recordings of the focus groups were transcribed and coded using NVivo software version 12 (QSR International Pty Ltd. Version 12, 2018, Victoria, Australia) [30]. The 3 phases of open, axial, and selective coding were followed according to the inductive approach in grounded theory [31]. In the first phase, 3 focus groups were openly coded by 2 authors (S.T. and M.B.) independently. This resulted in a preliminary coding scheme in which similar codes were clustered, and an initial hierarchy was applied. S.T. and M.B. reviewed and discussed this coding scheme until a full consensus was reached. Next, all 14 focus groups were divided between the same 2 researchers to complete the axial coding phase. New codes were added to the coding scheme if necessary and after consensus was reached. Finally, overarching themes were developed in the last phase to draw conclusions and to discover new information on help-seeking behavior patterns. The researchers agreed that data saturation was reached and that it was not necessary to perform additional focus groups. The 3 stages of the youth help-seeking and service utilization model of Srebnik et al. was used as a guiding theory to interpret the results (see Figure 1). This model consisted of 3 stages of youth’s help-seeking. It states that the decision to seek help and to turn to certain help sources is a result of (A) recognizing the problem, (B) predisposing, characteristics such as age and religion and, (C) barriers and facilitators. 

## 3. Results

A total of 14 focus groups were held with 71 participants. Adolescents had a mean age of 15.0 years (SD = 1.0). Table 1 provides an overview of the composition of the focus groups.

The results are structured within the three stages of the above mentioned help-seeking pathway [14].

### 3.1. Stage I: Problem Recognition

In general, the participants correctly recognized Loek (vignette 1) to have questions about intimacy and about feeling insecure but did not consider these issues as severe. On the contrary, the participants noticed the problems of Quin (vignette 2) to be severe:

*“Yes, and Loek* (vignette 1) *might think he has a big problem with his questions about his relationship and that he cannot go to his parents, while Quin* (vignette 2) *has way bigger problems”* (Female).

The way they weighed the severity of the problem seemed to influence their help-seeking behavior since the severity of the problems in vignette 2 made it more difficult for adolescents to decide what kind of help-seeking behavior to display. For this severe vignette, some adolescents mentioned to seek out one person they confided in, whereas others did not know what the person in this vignette should do or suggested more evasive behavior such as running away, seeking a way out by using drugs or transferring to a different school. 

*“With Loek* (vignette 1) *it was something that every general teenager could handle, that is something for which, if necessary, you could go to a website and talk to someone and then you could address how you should deal with this. But with Quin* (vignette 2) *that is something more serious, because if your home environment and your school environment are both disrupted, in those cases a person could seriously think about suicide because that person is fed up with it. That is far more serious than someone who is in doubt about whether they should have sex with someone”* (Female).

### 3.2. Stage II: Decision to Seek Help

Several factors that contributed to the decision to seek help were mentioned by adolescents. In all focus groups, the importance of a bond of trust with a help source was reported as a facilitator for seeking help: 

*“Everyone has this person who they trust, I think, where you can certainly go to. A friend, perhaps a mother or father, somebody you definitely trust”* (Female).

*“No, of course not in school, are you crazy, I trust no one in school”* (Male).

In addition, adolescents indicated it to be important for a source to be experienced in the area for which they were seeking help and that the source would have expertise on the matter, for example, with intimacy issues. In this respect, some adolescents reported to prefer sources of help being older, as they would be more experienced: 

*“No, primarily to my sister. I think it is easier when someone is older than you are because they have more experience. They know better how to handle some situations”* (Female).

*“For me, my friends would not be an option because we have the same age, and they are in the same boat. So, you can talk about your problems, that can be very nice, but I don’t think you will find solutions”* (Male).

However, some adolescents indicated to prefer little age difference with their source of help (i.e., peers).

The unfamiliarity of help sources was both mentioned as a possible facilitator and a barrier for seeking help. It was a facilitator when adolescents wanted to be sure their issues would not get back to family or friends because they did not want to be judged by them. For these help sources, the feeling of privacy played an important role in the process of opening up: 

*“I think it is nice that it is not with people who are close, because they might judge about your problem”* (Female).

*“With friends you are scared that other people in your environment hear about it”* (Female).

Unfamiliarity was a barrier when adolescents preferred a bond of trust with the source of help, someone who is more familiar with the situation or character of the person who seeks help: 

*“Yeah, but she (unfamiliar help source) does not know you personally”* (Male).

The topic of the issue was another factor contributing to their decision to seek help. It could create feelings of embarrassment, which would form a barrier to seek help for that issue, for example, the topic of intimacy specifically with parents: 

*“But if you really want to do that (kissing) then your parents might find you too young for it and then you hear about that”* (Female).

*“I would find that (intimacy) a bit inconvenient to discuss (with parents)”* (Male).

Some participants indicated they would not start a conversation about relationships or intimacy. Instead, they reported to expect help sources such as their parents to start this conversation and ask the adolescent about it: 

*“I would just discuss it with my parents. I think I would not bring it up myself, but if they ask how my relationship is going, then I would tell them”* (Female).

*“I think it should partly come from parents. If your parents do not start the conversation with you or do not talk about it with you, then you won’t initiate the conversations yourself”* (Male).

### 3.3. Stage III: Support Network and Service Utilization

Adolescents mentioned a variety of possible help sources within their support network or when utilizing more formal services; relatives (i.e., parents, siblings, grandmothers and grandfathers, aunts and uncles, cousins), internet, school environment (i.e., teachers and mentors), partners, friends, parents of friends, self-reliance, and other sources such as phone helplines, general practitioner, and youth health care professionals. The latter two were more often mentioned in the focus groups, where only girls participated, whereas internet was more often mentioned by boys as a possible help source to find solutions: 

*“I would go to my boyfriend”* (Female)

*“Friends or the internet”* (Male).

*”No, and sometimes you don’t want help and you want to take care of it yourself”* (Female).

*“If I were Loek* (vignette 1), *I would go to the general practitioner myself, I really would”* (Female).

*“Then you go to the general practitioner because I don’t expect them to give bad advice”* (Female).

A preference for informal help sources was visible in all focus groups. They indicated friends as their number one source of help, followed by parents, siblings, reliance on oneself, members from the extended family, and parents of friends. 

The topic of the issue would direct adolescents to a certain help source, such as school issues that were discussed with a mentor or parents. For the “mild” issues in vignette 1, participants often mentioned they would go to friends or search for answers on the internet. For the “severe” problems depicted in vignette 2, participants would seek out one person they confide in, for example, a mentor from school or a family member. 

*“Yes, for me, when it is about school, then you can discuss it with your parents or mentor. And if it’s about relationships, then you can discuss it with friends or a brother or sister, not really with parents”* (Female).

Varying views were found on using the internet as a source of help. Some adolescents indicated one might use the internet as an easily accessible source of help when you do not feel comfortable talking to someone about it, and when you have a specific or small question: 

*“Internet is helpful when you have one particular question….Yes, or when you don’t like to talk”* (Females).

*“Internet, I think you can really find something there. Just google”* (Male).

Internet was seen as especially useful when the topic for which help is sought caused feelings of embarrassment: 

*“Yeah, I think, for example, that if you have a problem, for instance, that you are pregnant or something like that, then I would first go to my best friend. But let’s say you find out that you have an STD, then I would google first, that is something for which you are ashamed of”* (Female).

Adolescents also mentioned the dangers of the internet, such as non-anonymous registrations of online data by youth health care. They stated that it would be hard to remove something from the internet once it is placed there, and they also emphasized that you never know who the person was that you were communicating with. That was the reason for some adolescents not to use websites or online help sources like group chats that are offered by health care providers. Some participants also would not use the internet for their questions because their parents could check their internet search history. The Internet was seen as impersonal and the participants stated you do not know whether the information you receive or find is correct.

*“On the internet, you hear that frequently, you never know who is behind it. What if there is some creep behind it… yeah you never know who it is”* (Female).

*“I think internet is less reliable compared to someone’s own experiences. I have more trust in my family than what some random person placed on the internet”* (Male).

*“Internet stays, it stays on the internet, and everybody can search for it on the internet and on the internet, you will find out for sure who it was who sent it, and that goes very quickly”* (Female).

*“You also have those group apps, or what’s it called, the youth health care also has a special forum. I would never do that”* (Female).

## 4. Discussion

In this qualitative study, there were 14 focus groups (N = 71 adolescents) to explore adolescents’ help-seeking behaviors for emotional and behavioral issues. In all focus groups, the issues in vignette one were correctly identified as surmountable, while the participants indicated that the person in vignette two was in need of serious help. Several contributing factors for seeking help were a bond of trust with a help source, the experience of a help source with the issue at hand, and the ability of a help source to start a conversation about intimate topics. A preference for informal help sources, as opposed to formal help sources, was clearly expressed by adolescents. When a mild issue would arise, adolescents mentioned friends and the internet as their first choice of help sources. When the problem would be more severe, the chosen help source was often a confided person, such as a mentor at school or a parent.

The main facilitator identified in this study to seek help from a certain help source was trust. This implies that a specific focus should be given to establishing a bond of trust between health care professionals and adolescents to stimulate help-seeking behaviors among formal help services. 

Adolescents stated they would not start the conversation about relationships and intimacy themselves, but they would discuss it when help sources, like parents, initiated this conversation, and also expect this from them. This highlights the importance of proactive and open communication by help sources about sensitive topics, like intimacy. Previous literature also demonstrated this since parent-adolescent sexual communication is associated with safer sex behavior among youth [32]. Interventions promoting help-seeking in adolescents should focus on the ability of parents and friends to open the discussion about sensitive issues.

The clear preference for informal help sources, especially friends, resonates with previous research where the majority of the studied youth do not seek professional help, but do seek help from their informal network, most commonly from peers [33]. Therefore, we recommend interventions that aim to promote help-seeking behaviors in adolescents to focus on peers as gateway providers to formal health care services. Peers should be aided in their ability to do so since research revealed adolescents’ health knowledge was often lacking, and they were not always aware of their role as gatekeepers to adult health providers [34]. Furthermore, a preference for seeking help with a confided person like parents or a school mentor was mentioned when severe problems would arise. This indicates that formal health services may be able to aid adolescents with severe problems through collaboration with parents or school mentors. 

Adolescents mentioned using the internet as either an easily accessible help source where answers to small issues and questions about health could effortlessly be found or as a help source for bigger issues that cause feelings of shame. Many participants stated the dangers of using the internet for seeking help, mostly with respect to privacy matters and not knowing who you were in contact with. This was in line with a review on internet use in youth where online privacy was a key issue for youth [35]. Our finding highlights the importance of youth health care professionals to make sure that adolescents are aware of accurate online help sources when finding answers to delicate questions. In general, it seems the internet must be seen as a source for information that is inquired additional to real-life informal help sources. Future research should further explore the use of the internet as a help source in adolescence since this help source was used for varying purposes.

More similarities than differences were found between the focus groups with boys and girls. However, several remarkable differences were identified. Female participants mentioned formal service utilization (e.g., general practitioner and youth health care) more often, compared to male participants. This in accordance with earlier research [36,37,38], where, for example, boys were less likely to consult and recommend professional help and more likely to recommend a self-help strategy compared to girls [37,38]. The finding that girls mentioned the use of the internet as a source of help less often was in contrast with previous research, where either no gender differences in health-related internet use were found or girls were found as more frequent users [35,39]. Gender differences in internet use need to be further explored in terms of gender-specific desires in internet use and ways to improve access to health information for both sexes. 

The strengths and limitations of this study should be considered when interpreting the results. By conducting a large-scale and thorough qualitative focus group study among six high schools, within different school levels and separate for boys and girls, an in-depth understanding of adolescents’ views towards help-seeking behavior was gained. Several methodological considerations should be mentioned. To include participants within the high schools, a convenience sample was taken. The fact that classes were informed about the study, and adolescents could apply when they were interested may have hampered the generalizability of our results. For example, when the non-response was particularly high among vulnerable adolescents due to emotional or behavioral issues. Second, certain adolescents may have dominated the focus group discussions, and adolescents may have expressed socially desirable answers. The researcher conducting the study, however, was trained to minimize these risks. 

Another strength of this research was that it discussed daily life issues and more serious issues from the point of view of adolescents in the form of two vignettes. The use of the vignettes gave the ability to discuss sensitive topics in a structured and non-threatening manner, compared to discussing personal experiences of the participants. However, the use of vignettes may have elicited different views than real-life situations would have [27]. Another strength of the study was the use of gender-neutral names in the vignettes, thus that gender bias could be prevented. All focus groups took place during teaching hours at the school in which the participants were enrolled. This was facilitating for the participants, as it took minimal effort in terms of traveling and time. However, the location could have influenced the sense of privacy the participants had. To minimize this possibility, an effort was taken to carefully select a secluded room where no one could look inside, and teachers were asked beforehand not to enter the room. Furthermore, the focus groups were held separately for boys and girls. Besides exploring the possible differences in help-seeking behaviors between boys and girls, this was done to create an environment in which all participants felt comfortable to speak their mind. It could be interesting also to include mixed focus groups in future studies, to look at the influence of mixed groups in discussing certain topics. 

## 5. Conclusions

In conclusion, this large focus group study demonstrated that adolescents preferred to seek help with a person they can confide in when the issue is experienced as severe. A bond of trust with the help source as well as a help source demonstrating his/her expertise was regarded as the main facilitators for seeking help among adolescents. Our findings support the importance for help sources, in particular, formal service providers, to be trained to invest in a bond of trust with adolescents, in communicating their expertise, and in starting a conversation about topics that could evoke feelings of embarrassment for adolescents, such as intimacy-related topics. Furthermore, this study highlights the importance of collaboration between peers, parents, school mentors, and youth health care providers in dealing with adolescents’ emotional and behavioral problems, particularly when it concerns severe issues.

## Figures and Tables

**Table 1:** Structure of the 14 focus groups with adolescents aged 13–18 years.

Focus Group	Number of Participants	Gender ^1^	Age Range	Educational Level ^2^
A	6	F	13–15	PVE
B	6	M	13–15	PVE
C	6	F	14–15	PVE
D	5	M	14–15	PVE
E	6	F	15–17	SSE
F	6	M	15–18	SSE
G	6	M	15–16	PVE
H	6	F	14–17	PVE
I	5	F	15–16	SSE
J	3	M	16	SSE
K	6	F	14–16	PVE
L	3	M	14–15	PVE
M	4	F	15–16	SSE
N	3	M	15–16	SSE
Total	71 (32M, 39F)	7M, 7F	Mean = 15.0 (SD = 1.0) range 13–18	8 PVE, 6 SSE

^1^ M = males, F = females. ^2^ PVE = Pre-vocational education, SSE = Senior secondary education.

## Appendix A

VIGNETTE Loek

Loek* is in the fourth year of senior secondary education at a school in Amsterdam. Loek feels well, has fun at school and obtains high grades. There is a good atmosphere in class, for example no one is being bullied. Also in the weekend, Loek has enough to do, like working at the supermarket or playing sports at the athletics club. At home it is also fine, but Loek is increasingly feeling like keeping everything to one’s self and sharing very little with for example parents. Recently, Loek started dating, which is all very new and is accompanied by a lot of insecure feelings. Loek has many questions about this relationship about, among other things, kissing and sex and how to deal with that in a relationship. Loek does not dare to discuss this with parents, because of the shame Loek feels, because how do you go about discussing something like that and how do you deal with the insecure feelings?

*Loek is a unisex name in the Netherlands, which enabled both sexes to identify with the vignette

VIGNETTE Quin

Quin* does not feel well, because school is not going well and a number of students are behaving annoyingly towards Quin. There have been times when Quin truanted and did not feel like going to school. It is also not going well at home, because there are a lot of arguments. Quin is getting sick more and more often, Quin does not know what to do.

*Quin is a unisex name in the Netherlands, which enabled both sexes to identify with the vignette

## Appendix B

All Questions used as prompts during the focus groups

1. Is this a good description of a peer?
a. Why/why not?
2. Do you think Loek/Quin needs help for specific questions?
a. (prompt: for example about feeling insecure)
3. If Loek/Quin would want help, where would you seek help if you were Loek/Quin?
4. How would you prefer to receive help?
a. On the internet
i. (how would the internet help?)
b. In books
i. (how would books help?)
c. At school
i. (how would school help?)
ii. Who would you go to in school?
d. Family
i. (how would family help?)
ii. Who would you go to in your family?
e. Peers
i. (how would peers help?)
f. From an unknown professional who regularly visits school
i. (how would this person help?)
g. From a certain organization
i. (how would this organization help?)
h. From the youth health care?
i. (how would the youth health care help?)
5. What do you think you peers have many questions about?
